# A New Rehabilitation Tool in Fibromyalgia: The Effects of Perceptive Rehabilitation on Pain and Function in a Clinical Randomized Controlled Trial

**DOI:** 10.1155/2016/7574589

**Published:** 2016-01-13

**Authors:** Teresa Paolucci, Carlo Baldari, Manuela Di Franco, Dario Didona, Victor Reis, Mario Vetrano, Marco Iosa, Domenica Trifoglio, Federico Zangrando, Ennio Spadini, Vincenzo Maria Saraceni, Laura Guidetti

**Affiliations:** ^1^Physical Medicine and Rehabilitation Unit, Policlinico Umberto I, “Sapienza” University of Rome, 00185 Rome, Italy; ^2^Department of Movement, Human, and Health Sciences, Foro Italico University, Rome, Italy; ^3^Department of Internal Medicine and Medical Specialities, Rheumatology Unit, “Sapienza” University of Rome, 00185 Rome, Italy; ^4^Research Center for Sport, Health, and Human Development, University of Tras-Os-Monte and Alto Douro, Vila Real, Portugal; ^5^Department of Orthopaedics and Traumatology, Sant'Andrea Hospital, “Sapienza” University of Rome, 00185 Rome, Italy; ^6^Clinical Laboratory of Experimental Neurorehabilitation, Fondazione Santa Lucia IRCCS, Rome, Italy; ^7^Physical Medicine and Rehabilitation Unit, Salus infirmorum, S. Filippo Neri Hospital, Rome, Italy

## Abstract

*Introduction*/*Objective*. Fibromyalgia might benefit from a specific tactile and proprioceptive rehabilitation approach. The aim of this study was to perform a randomized controlled trial to determine the efficacy of perceptual surfaces (PS) and physical exercises with regard to chronic pain and physical function in fibromyalgia compared with a control group.* Methods*. Data from 54 females (18–60 years old) with a diagnosis of fibromyalgia and scoring >5 on the visual analog scale were divided into 3 groups and analyzed: group treated with perceptual surfaces (PS-group), physical exercises group (PE-group), and control group (CG). The Fibromyalgia Impact Questionnaire (FIQ), Health Assessment Questionnaire (HAQ), and Fibromyalgia Assessment Scale (FAS) were administered at baseline (T0), at the end of the treatment (T1) (after 10 rehabilitation sessions over a 5-week period), and at the 12-week follow-up (T2).* Results*. The PS-group experienced a statistically significant improvement versus the CG in FAS and HAQ scores. Good efficacy with respect to pain and function in the PE-group compared with the CG in terms of FAS, HAQ, and FIQ scores was observed. The adherence ratio was 86% for the PE-group and CG and 90% for the PS-group.* Conclusions*. According to the results, the PS are as promising as the physical exercises, since results were similar.

## 1. Introduction

Fibromyalgia (FM) is a condition of generalized, chronic, widespread pain that is usually accompanied by fatigue, sleep disturbances, psychological and cognitive alterations, joint rigidity with muscle stiffness, and tenderness [1, 2]. FM is a prototypical form of central sensitization syndrome that affects the dysregulation of mechanisms that normally govern pain sensation [2] and must be treated in a multidisciplinary fashion, which entails physical exercise, multimodal cognitive behavioral therapy, and pharmacological therapy [3–5].

Patients with FM spend more time engaging in sedentary activities [6, 7]; thus, physical exercises (PE) have a direct positive impact on joint rigidity, muscle stiffness, widespread pain and tenderness, and fatigue and secondary positive effects on cognitive dysfunction. Evidence-based aerobic exercise programs focus on increasing strength and flexibility, but they can not have high-intensity or be performed too frequently [8–11]. Low-impact exercise programs and the constant ability to individualize the protocol are crucial in ensuring optimal adherence to such regimens to promote a shift to a more physical lifestyle with a good attitude [12]. Isometric contractions are not recommended, because they increase muscle pain, discouraging further exercise in someone who already engages in little physical activity [13]. Aquatic exercise training appears to be a viable alternative [14–16], but adequate recovery strategies must be applied, and the patient should be motivated to continue these exercises after participating in such a program [17, 18].

Other alternative complementary exercises and proprioceptive approaches exist in the treatment of chronic pain, also in FM [18–22]. The practice of Tai-Chi, for example, has been associated with improvements in strength, balance, and flexibility in patients with FM [23, 24].

Generally, exercise activates the endogenous opioid and adrenergic systems but does not consistently mitigate pain in FM patients [25], possibly due to sensitization of the primary afferent pathways or the dysfunction of endogenous systems that modulate afferent activity in FM and the overall increase in sensitivity [26]. Pain has seemingly infinite interindividual variability, wherein a complex relationship exists between proprioceptive capacity, tactile acuity, pain intensity, and cortical organization [27, 28]. Thus, as reported, a specific tactile and proprioceptive rehabilitation program, comprising somatosensory stimuli to the painful area, patient-specific perceptive exercises, and motor imagery, can reduce pain and sensory dysfunction [27–29]. Abnormal cortical excitability in patients with FM affects the nociceptive-specific cortical matrix and second-order perceptual matrix [29, 30] and underlies the dysregulation of emotionality and body homeostasis in the insula [31, 32]. Based on the changes in an FM patient's matrix perception, we propose a new perceptive rehabilitation tool—based on the use of perceptual surfaces (PS)—that creates a new flow of sensory-motor afference throughout the body in the perceptual matrix that might generate positive feedback with respect to pain threshold and sensation [1, 2].

The aim of our study was to determine the efficacy of rehabilitation with PS and PE in reducing chronic pain and improving physical function in female patients with FM versus a control group.

## 2. Materials and Methods

The research protocol was reviewed and approved by the ethics committee (number 2547-720/2012, clinical trial number 02472093) of “Sapienza” University of Rome (Italy). From May 2012 to May 2013, 88 potentially eligible outpatients were screened, 62 of whom met the inclusion criteria and gave their written informed consent after receiving detailed information about the aims and study procedures per the Declaration of Helsinki. The study was carried out at the physical medicine and rehabilitation unit, in collaboration with the rheumatology center, of the Policlinico Umberto I in Rome (Italy).

The inclusion criteria were satisfaction of the American College of Rheumatology (ACR 1990 and 2010) criteria, which include experiencing widespread pain for more than 3 months and pain with 4 kg/cm^2^ of pressure on 11 or more of 18 TPs (in every case, the diagnosis of FM had been established by the patient's rheumatologist); age of 18 to 60 years; a score of >5 on the visual analog scale (VAS), in the last three months; tenderness of at least 2 of the 4 tender points on the back; and baseline condition of sedentary lifestyle with no or irregular physical activity.

The exclusion criteria were the presence of concomitant autoimmune diseases, psychiatric disorders (as a diagnosis of major depression), or other causes of chronic pain; other diseases that prevented physical loading; severe scoliosis or kyphoscoliosis; surgery of the spine; vertebral fractures; sciatic pain; tumors; and enrollment in another type of physical therapy program. Patients were excluded if they had comorbidities, such as cardiovascular risk factors, previous myocardial infarction, lower extremity arterial disease, major neurological problems, diabetes, gastrointestinal disease, chronic respiratory disease, kidney disease, and poor vision. The pharmacotherapy regimen must have been stable for at least 3 months before the patient began treatment: acetaminophen up to 3 g/day, tramadol up to 200 mg/day, and pregabalin up to 150 mg/day.

### 2.1. Study Design and Data Collection

**Figure 1:**
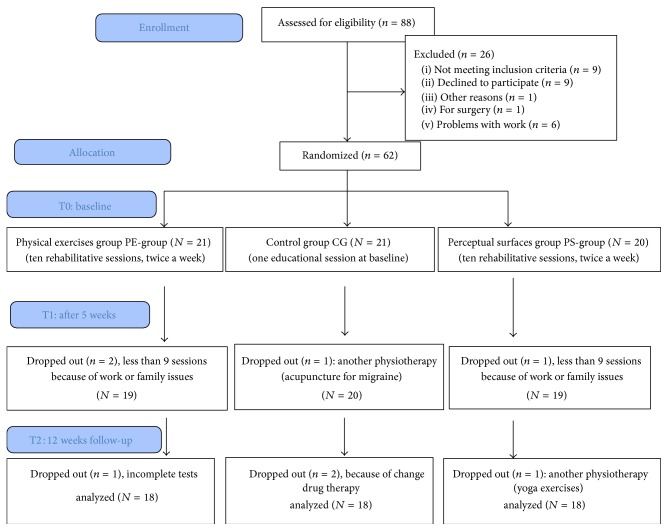
Flowchart.

A total of 88 female patients with FM were enrolled, 62 of whom were randomly allocated into 3 treatment groups (simple randomization) with the different rehabilitation programs: perceptual surfaces group (PS-group, *n* = 20), physical exercises group (PE-group, *n* = 21), and control group (CG, *n* = 21). At the end of the study, data from 54 patients were analyzed (18 patients per group) (Figure 1).

With regard to allocation concealment, a physiatrist identified the patients who were sent by the rheumatologist to confirm the inclusion and exclusion criteria and eventually obtain signed informed consent. Then, the patient was asked to draw a sealed envelope from a box that contained a piece of paper with the group assignment, which was concealed until the envelope was opened. Initially, 66 envelopes were in the box (according to the computed sample size increased of 10%), with an allocation ratio of 1 : 1 : 1, that is, 22 envelopes for each group. The envelopes were then consigned to a researcher who did not perform the assessment.

Outcome assessments were performed for each group before treatment (T0), at the end of treatment (T1) (after 10 rehabilitation sessions over a 5-week period, held twice a week, with each session lasting 60 minutes), and at the 12-week (T2) follow-up. Every week, between T1 and T2, the physiatrist telephoned patients (of all the three groups) for clinical interviews regarding changes in their chronic pain. Further, these phone calls were made to ensure continuity of the patient's physical activity, as recommended during rehabilitation. Patients were given the opportunity to call the group physiatrist during the follow-up at any time in case of exacerbation of pain or other problems that were related to FM to ensure the patient's continuity in managing his care.

The study was a randomized controlled trial (RCT): to reduce the potential for bias, all subjects were evaluated by the same blinded researcher at T0, T1, and T2.

### 2.2. Evaluation of Fibromyalgia

The* Fibromyalgia Impact Questionnaire* (FIQ) comprises 3 sections—function, impact, and symptoms—that are summed to generate an overall score. The first section contains 10 subitems and focuses on the patient's ability to perform daily tasks that involve the major muscles (e.g., cooking, cleaning, walking, shopping, homemaking, socializing, and mobility). The next 2 items ask patients to circle the number of days in the past week on which they felt good and the number of days that they missed work. The last 7 items assess (i) the ability to do one's job, (ii) pain, (iii) fatigue, (iv) morning tiredness, (v) stiffness, (vi) anxiety, and (vii) depression. The total FIQ score is calculated by adding the following 10 items: (i) the physical function score, (ii) the number of days feeling good, (iii) the number of work days missed, (iv) the ability to do one's job, (v) pain, (vi) fatigue, (vii) morning tiredness, (viii) stiffness, (ix) anxiety, and (x) depression. The FIQ score ranges from 0 to 100, with 100 indicating the worst possible score due to FM. The italian version of the questionnaire was used [33]. A 14% change in the FIQ total score is clinically relevant [28].

*The Fibromyalgia Assessment Status (FAS) *is a simple and rapidly implemented index, consisting of a pain map, called the Self-Assessment Pain Scale (SAPS) (on which the patient is asked to indicate how much pain he suffered in the previous week in 16 areas of the body, with scores ranging from 0 to 3), and 2 scales that evaluate fatigue and quality of sleep, with scores ranging from 0 to 10. The FAS allows physicians to obtain reliable information concerning the course of the disease, and it is sufficiently sensitive to alert them to deterioration in the patient's condition [34].

*The Health Assessment Questionnaire (HAQ)* is a 20-item self-administered questionnaire that determines the difficulty in performing 8 daily activities: (i) dressing and grooming, (ii) getting up, (iii) eating, (iv) walking, (v) hygiene, (vi) reaching, (vii) ability to grip, and (viii) outside activities. For each item, patients are asked to rate the level of difficulty that they experienced over the previous week in performing these activities on a 4-point scale, ranging from 0 (no difficulty) to 3 (unable to perform). The final HAQ score is the average of the 8 categories and ranges from 0 to 3; higher scores reflect greater levels of disability [35].

#### 2.2.1. The Rehabilitation Program

**Figure 2:**
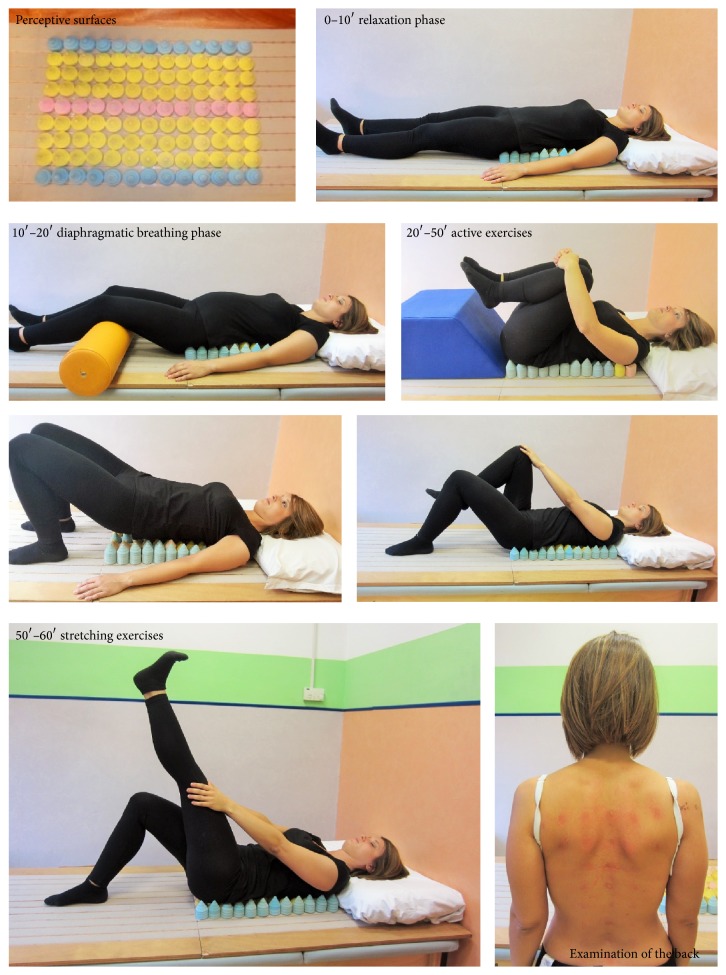
Perceptual surfaces and execution of a rehabilitation session.

*(A) Perceptive Rehabilitation Program*. Perceptive surface group (PS-group) received a treatment that, as described by Morone and colleagues [36–38], is a therapeutic approach based on the interaction between the patient's back or painful area and a support surface, composed of small latex cones with various dimensions (height: 3–8 cm; base diameter: 2–4 cm) and elasticities. The inferior bases of these cones are applied to a rigid wood surface using elastic strips; normally, over 100 cones are used for each session (Figure 2).

Patients were asked to lie down supine on the surface that was formed by the smoothed apex of these cones, creating reaction forces against the patient's weight that were generated by the interaction with the cones. The cones varied in elasticity (20%, 40%, and 60%), due to the malleability of the latex material. The rehabilitation protocol involved 2 sessions per week (for 5 weeks), for a total of 10 sessions, plus an introductory session that lasted 60 minutes. A brief educational session during the first PS rehabilitative visit was conducted: the scope was aimed to teach the patient how to cope with FM pain and to practice the exercises independently to improve rehabilitation and compliance with the treatment. This instruction was provided by a physician who specialized in physical medicine and rehabilitation; the other individual sessions were conducted by a physical therapist.

1. In the first session,* a sensory-motor evaluation* was performed, in which medium-elasticity cones were used as a reference of the midline, with the environment remaining neutral. Patients were asked to relax, find the most comfortable position, and breathe normally (10 minutes for the relaxation phase and 10 minutes for the slow diaphragmatic breathing phase).
2. Then, the patients performed* active exercises*, consisting of tactile and proprioceptive tasks with increasing difficulty in perceiving the areas of support. The patients were asked to indicate the surface of the body that was in contact with a particular area, to describe and count the number of cones, to check the distribution of the load on the bed and correct it, and to pay attention to posture. The patient had to evaluate what he perceived and felt, especially if the load was distributed uniformly and symmetrically with respect to the trunk midline. Patients who alternated spontaneous breathing with diaphragmatic breathing exercises were relaxed. During the training sessions, patients performed* cognitive-perceptive* tasks to improve the perception of their trunk and, in particular, its midline, supervised and guided through the session in their motor tasks by a physical therapist. Also, at the end of each session, the physical therapist examined the interaction between the skin on the back and the surfaces that relieved the hyperemic area on the patient's back.
3. Specifically, the 30 minutes of active exercise included exercises of the bridge, exercises for crosslimb coordination, and bringing an egg that was held by the knees to the chest. Each exercise was performed 10 times in 3 sets, with 3 minutes of rest between sets and no placement on isometric hold.
4. In subsequent sessions, cones with varying elasticities were positioned by the therapist to improve the symmetry of contact between the surface and the patient's back, taking into consideration the hyperemia in the previous session. The objective of this step was to obtain* reafferent* information from the trunk and body, the positions of which were altered in FM, using elements of motor imagery [38–41].

Each phase of the session ended with 10 minutes of global stretching of the upper limbs and lower limbs. An example of a sequence with perceptual surfaces is shown in Figure 2.

*(B) Physical Exercise Program*. Physical exercises group (PE-group) received a conventional treatment based on a program comprising 10 1-hour sessions, held twice a week (over a 5-week period), with 4 patients per group. A brief educational session on FM was conducted by a physiatrist to enhance the patients' knowledge of FM and improve their self-care skills to increase their independence with regard to their pain management. The importance of practicing physical activities properly, perceiving one's body and emotions, and sharing experiences with other group members was the focus of the rehabilitation program.

A physiotherapist oversaw the rehabilitation sessions. The types of exercises included low- to moderate-impact aerobic training (starting from 40% of heart rate (HR) to a maximum of 50% to 60% of the age-adjusted predicted maximum heart rate); walking in a circle, alternating with periods of going up and down the stairs (3 steps for 10 minutes) for a total of 20 consecutive minutes [42]; and posture exercises for the back and proprioceptive exercises for the trunk in the supine position to improve axial stability, including diaphragmatic breathing. Heart rate was monitored on a heart rate monitor, to respect the fixed threshold.

1. *Exercises in the Supine Position on a Mat*. The subject was asked to (i) bring his foot to the head, alternating movements of the feet; (ii) bend the right knee and then the left and crawl on the heel pad, alternately stretching one leg after the other; repeat the previous exercises with both legs simultaneously; (iii) keep the knees bent with the heels on the ground, bringing the arms to the knees, with the palms facing inward and keeping the head resting on the ground on the occiput; (iv) perform retroverted and anteverted excercises for the pelvis with the knees flexed and heels on the ground; (v) load, first flexing the right knee to the chest and then the left and then bringing both knees flexed to the chest and embracing them with the arms (neutral rotation of the hip); (vi) load, first by flexing the right knee to the chest and then the left, combined with external rotation of the hip; and (vii) bend both knees to 90°, associated with hip flexion of 90°, holding this position for 6 seconds. These exercises were repeated with the column on a cylinder of soft material to stimulate proprioception.
2. *Exercises in the Quadruped Position*. This step involved a retroverted pelvis with a flexed trunk position and anteverted pelvis with trunk extension (engaging the abdominals, as if pulling the navel toward the spine and around one's back toward the ceiling, allowing the head and neck to fall naturally between the arms).
3. *Exercises in Upright Position*. The subject had to first keep his knees and back bent slightly against the wall, then slide down with his back by bending his knees while maintaining contact with the wall, and return to the starting position. These actions were repeated with a ball between the back and the wall. The patient was asked to keep his arms at his sides and the hands on the wall. Heel raises: the subject was asked to stand evenly on both feet and slowly move the heels up and down.
4. *Exercises While Sitting on a Ball*. The subject was asked to sit on a ball with his hips and knees bent 90° and his feet resting on the floor, slowly raise an arm over the head, and lower it, alternating the right and left sides. To finish, he was asked to slowly raise and lower each heel, alternating sides. Then, the subject was asked to slowly raise one heel and the opposite arm over the head, alternating with the opposite arm and heel. While marching, the patient had to raise 1 foot slowly 2 cm off of the floor, alternating sides.
5. *In the Prone Position*. The subject placed a ball in front of his pelvis and raised both lower limbs. As if kicking, the subjects repeatedly raised and lowered the 2 lower limbs alternately. The subject first tried to stabilize his posture and then slowly raise the other lower limb. This exercise was performed 10 times for 10 seconds each.
6. *In a Circle, Exercises in Pairs with a Ball*. Back to back, with the knees slightly bent and feet 20 cm apart, the subject rotated his torso, first to the right and then to the left, passing the ball to his partner.

Each exercise was repeated 10 times (3 sets of 10), with a resting period of at least 3 minutes between sets. All sessions ended with stretching exercises, diaphragmatic breathing exercises, and relaxation.

**Figure 3:**
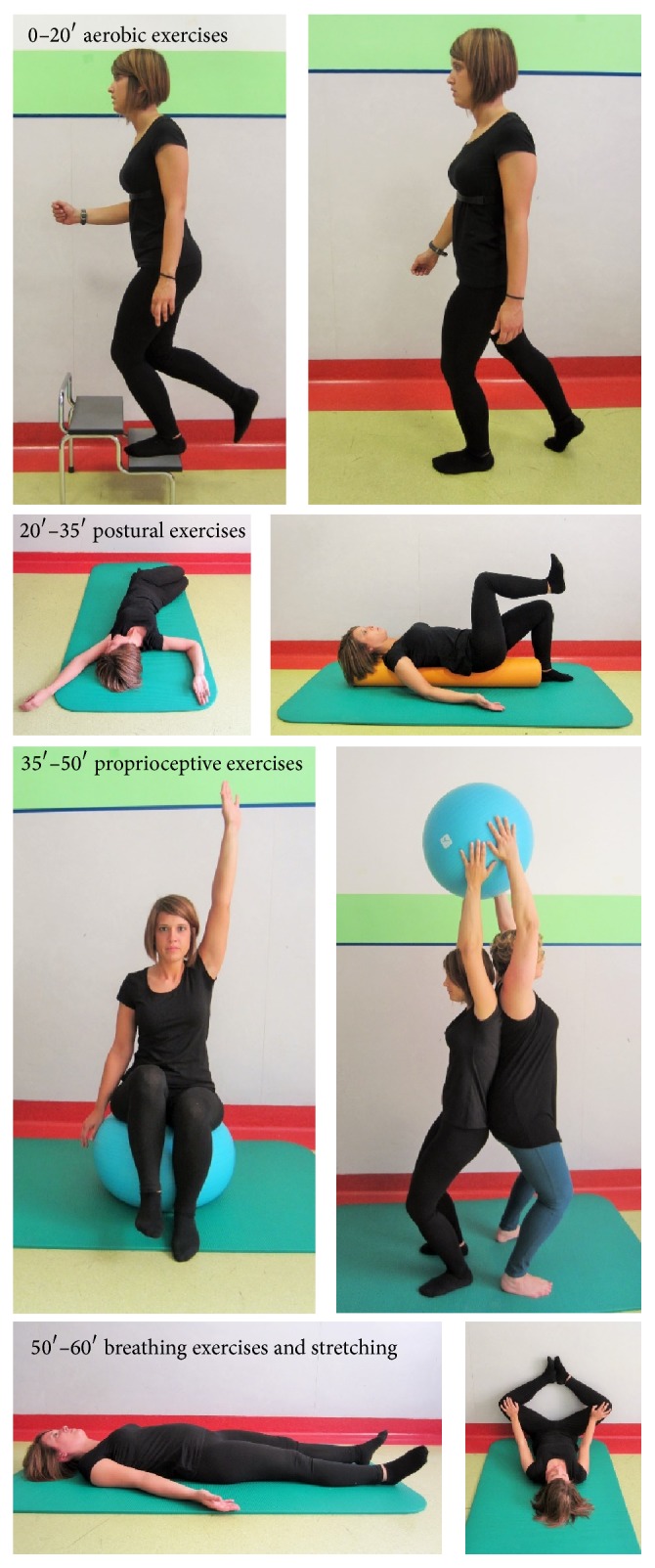
Execution of a session in the physical exercises group.

An example of this sequence in the physical exercises group is shown in Figure 3.

*(C) Control Program*. The physiatrist conducted 1 brief educational session on FM session that lasted for 1 hour for each patient to teach simple breathing exercises and relaxation techniques and careful stretching exercises (such as those in the TG) to perform at home, at least twice per week for 1 hour each time, to respect ethical recommendations. The control group was asked to continue their routine lifestyle during the study period.

### 2.3. Sample Size and Statistical Analysis

Continuous measures were expressed as mean and standard deviation and analyzed between groups by analysis of variance. For noncontinuous measures, such as clinical scores, median values and quartiles were computed, and data between groups were analyzed by Kruskal-Wallis test, followed by post hoc comparisons by Mann-Whitney *U* test after adjusting the threshold of significance to 0.025 by Bonferroni correction. Friedman analysis was performed for same-group comparisons. Upper and lower bounds of the 95% confidence interval were computed by Monte Carlo simulation of 1000 random samples. Effect size was computed in terms of Cohen's d. All data were analyzed using IBM SPSS for MAC v. 21 (IBM SPSS Inc., Chicago, IL).

Sample size was calculated starting from preliminary data related to the scores of FIQ of 5 patients treated with PS and those of 5 patients performing CG (using the online sample size calculator software developed by DSS Research, https://www.dssresearch.com/). The minimum number of patients to enroll for having a significant difference between the two groups was evaluated comparing means and standard deviations of FIQ scores at T1. For this analysis, alpha-level was set to 1% for considering Bonferroni correction (because the study design involved 3 groups and not only two) and beta-level to 10% for obtaining a power of analysis of 90%. Following this approach the resulting sample size for each group was of 16 subjects. A minimum of 20 subjects for each group was determined to take into account an estimated dropout rate of 20% (20 *∗* 0.8 = 16 subjects).

## 3. Results

A total of 88 patients were enrolled, 62 of whom were randomly assigned to the 3 groups (see Figure 1, flowchart). During the 5-week treatment period, 4 subjects dropped out (2 PE-groups, 1 PS-group, and 1 CG), and during the follow-up 4 subjects dropped out (1 PE-group, 1 PS-group, and 2 CG). Three patients performed fewer than 9 sessions due to work or family issues, 2 changed drug therapies, 2 performed another physiotherapy (acupuncture for migraine at T1, yoga exercises at T2), and 1 submitted incomplete tests. No patient discontinued the rehabilitation due to acute exacerbation of pain. At T2, data on 54 patients—18 from each group—were analyzed.

At baseline, the 3 groups were homogeneous with regard to age, height, weight, body mass index, and VAS score, as shown in Table 1. Also, the median scores for the 3 clinical scales did not differ significantly at baseline (T0).  Table 2 summarizes the results for the 3 outcome measures that differed significantly between T1 and T2. In the post hoc analysis, FAS (T1: *p* = 0.003, T2: *p* = 0.017) and HAQ (T1: *p* = 0.006, T2: *p* = 0.001) scores were significantly lower in the PS-group versus CG at both secondary assessments, whereas FIQ differed only at T2 (*p* = 0.004) (T1: *p* = 0.044, not significant for Bonferroni correction of threshold of significance).

**Figure 4:**
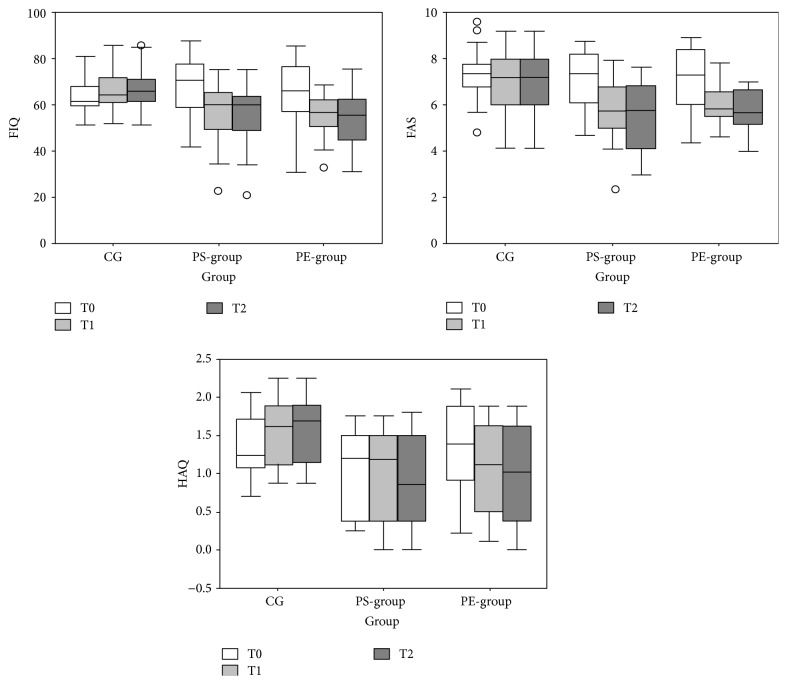
Box and whiskers plots of FIQ (Fibromyalgia Impact Questionnaire), FAS (Fibromyalgia Assessment Status), and HAQ (Health Assessment Questionnaire) scores for the CG (control group), perceptual surfaces group (PS-group), and physical exercises group (PE-group) at the 3 assessments (T0, T1, and T2). The boxes show the lower quartile, median (middle line in box), and upper quartile values. The whiskers represent the most extreme values 1.5 times the interquartile range from the ends of the box, and the circles represent data with values beyond the ends of whiskers.

In the PE-group, FAS (T1: *p* = 0.004, T2: *p* = 0.003), HAQ (T1: *p* = 0.010, T2: *p* = 0.002), and FIQ (T1: *p* = 0.003, T2: *p* = 0.002) scores differed significantly at both assessments from those of the CG (Table 2 and Figure 4). There were no significant differences between the PS-group and PE-group at any assessment. Within-group comparisons revealed that all 3 outcome measures declined significantly in the PE-group (see Table 2). For the PS-group, these reductions were significant only for FIQ and FAS. Conversely, the CG experienced small but significant increases in FIQ and HAQ scores but not FAS.

Patients did not change drug treatments during the study period.

## 4. Discussion

The most notable result of our study was the reduction in pain in female FM patients: PS had good short-term efficacy when compared with the CG. In the post hoc analysis, PS-group had significantly lower scores than controls, but the differences in FIQ scores were significant only at T2, most likely because the mechanisms of PS, based on proprioception to modify pain thresholds, are slower, consistent with other studies on chronic nonspecific low back pain [37–41]. As Bennett et al. underlined a 14% change in the FIQ total score is clinically relevant: in the present study [28], changes in the PS and PE groups surpassed this 14% change, so they have a clinical meaning as well.

Further, the effects at the end of treatment were maintained at the follow-up in both rehabilitation groups with regard to improved physical function and reduced pain. No significant differences were noted between the PS-group and PE-group at any assessment. PS-group was as effective as PE-group in FM patients, although the ability to perform daily tasks differed from controls at both assessments for the PE-group but only at the follow-up in the PS-group. Autonomy in managing activities of daily living and functional recovery also improved, paralleling the reduction in chronic pain: in the PS-group, pain declined significantly in a more slowly period than in the PE-group and continued to improve after suspension of the rehabilitation treatment. It could also be because the PE-group session was guided by the physical therapist as a group and the other individually.

This result might explain why the recovery of autonomy in daily life in the PS-group was significant only at T2 versus the CG.

We hypothesize that, per Melzack's theory, the new “perceptual experience” with no pain through PS modulates the body-self neuromatrix, replacing the previous experience “movement/perception = pain.” The output patterns from the neuromatrix generate the multiple dimensions of pain experiences and concurrent homeostatic and behavioral responses [27, 32]. As a possible cause of changes, it has to be considered as the physical therapist guided individually to the patient in PS-group. The so called therapeutic alliance has been concluded to have a huge impact on therapeutic intervention: therefore, this one to one relationship could be a better explanation for the findings in the PS-group [43].

Also, we support the hypothesis that tactile and proprioceptive rehabilitative stimuli promptly enhance motor-sensory control [36, 39, 40]. Chronic pain, as in FM, alters the real-time body schema, and motor-sensory alterations are reflected by increased activity in the prefrontal cortex, in which a motor-sensory conflict is generated [41]. Moreover, the real-time body schema and motor image bridge perception and memory as perception and motor control. If an image is changed, the perception is altered and conditioned by the image. Body image is disrupted in people with chronic pain disorders, and the consciousness of our body depends on internal maps that are continually modulated by somatic and proprioceptive inputs [39].

The brief educational intervention in the PE-group and PS-group on the importance of having correct motor habits and the rule of the perception of pain improved the adherence to rehabilitation: the adherence ratio was similar for the physical exercises group versus the perceptive group.Also, in the PE-group, sharing with other group members broke the pattern of fear avoidance, nearly recreating a path of self-help group [44–46].

For example, certain exercises in the PE-group have been proposed to facilitate exchange, such as exercising in a circle in pairs with a ball. Between groups, in the PS-group, only the FIQ and FAS scores fell significantly—not the HAQ score—versus the PE-group. The HAQ is based on the evaluation of daily activities: the PS-group did not work directly about these items, except in the first session. Instead, PE-group, as in the first lesson as in all other sessions, worked directly about these items.

The low- to moderate-impact aerobic training in the PE-group, with the brief educational intervention (active pain management strategies proposed in the first session and following) was effective in our FM patients, regardless of the severity of disease, consistent with other studies [12, 16, 17, 22].

Even if physical exercise is frequently recommended in the management of FM, the type (aerobic, strength, flexibility, or other proprioceptive exercises), intensity, duration, and frequency of exercise and possible adverse effects, such as increased pain and fatigue, must be identified to avoid significant dropout [44].

In our study, the overall dropout rate was 12.9%, far below what was initially established (20%) in the sample size calculation, because our female FM patients had good treatment compliance. The dropout rate was similar between groups, suggesting similar compliance between them. No dropouts were due to changes in drug therapy, previously determined by a rheumatologist, for increasing in pain.

Also, the patient could concentrate on creating a new experience (no pain) with the body through exercise and experiencing the body's sensations.

In our experience, the relaxation phase at the beginning of rehabilitation approach is paramount to harmonize with the body and mind. For example, all complementary and alternative medicine rehabilitation (CAM) therapies, which often propose approaches with group exercises, unlike more traditional rehabilitative interventions, emphasize the relaxation phase and contact with oneself. The use of CAM exercises is widespread and increasing worldwide [47]. It has been suggested that nontiring physical exercise, mind-body exercise, and certain types of relaxation therapy increase the tolerance to pain improving the overall quality of life of FM patients [50]. Also, CAM therapy, in contrast to more conventional rehabilitation approaches, continuously monitors what the patient is doing by listening to the body and promotes teamwork. This method, especially Tai-Chi, is effective for FM patients [24]. Nevertheless, Canadian guidelines suggested caution regarding CAM therapy in FM [48].

There are some weaknesses in the design of this study. Longer follow-up periods should be considered, perhaps up to 12 weeks. However, our purpose was to determine the efficacy of a new approach with PS in treating FM, which is effective in the short term. Further studies could also integrate PS treatment with other rehabilitation approaches.

Also, we did not assess the state of depression symptoms with specific scales during the treatment. Future research should study the reduction in pain in FM during rehabilitation while examining the impact of pain on depression and vice versa. Further, we did not perform a sub max exercise test, because our subjects had a sedentary lifestyle at baseline and did not have major cardiovascular risk factors [42]. We are interested in extending the study to an FM population with an aptitude for physical activity at baseline that is more pronounced in motor habit.

The efficacy of PS suggests that, in patients with FM, it could be contemplated an incorrect perception of pain. In addition, exercises that promote relaxation and self-perception through awareness of one's body are effective. As emphasized in the literature, relaxation as a single therapy should not be applied; instead, cognitive behavioral therapy, combined with the physical exercises, is strongly recommended [49, 50].

## 5. Conclusions

Perceptual surfaces are efficacious in treating female patients with FM, similar to physical group exercises, improving physical function and mitigating pain. Both PE and PS (both groups with success) were supervised and guided from physical therapist and not the control group: in the PS-group the patient and physiotherapist had a one to one relationship. Future studies should evaluate the synergistic effects of PS and physical exercise as in PE-group in FM patients.

## Figures and Tables

**Table 1:** Group comparisons at baseline. Mean and standard deviations are reported. *p* values were computed using analysis of variance for continuous measures and Kruskal-Wallis analysis for VAS scores.

Baseline parameter	PS-group	PE-group	CG	*p* value
Age [years]	49.3 ± 11.1	50.4 ± 8.6	51.3 ± 9.0	0.814
Height [m]	1.61 ± 0.06	1.62 ± 0.08	1.67 ± 0.08	0.055
Weight [kg]	66.4 ± 18.8	64.6 ± 9.2	68.6 ± 14.9	0.715
Body mass index [kg/m^2^]	25.5 ± 6.3	24.7 ± 3.7	23.8 ± 5.0	0.615
VAS	7.7 ± 1.3	6.9 ± 1.7	7.2 ± 1.8	0.482
Duration of FM [years]	6.23 ± 3.55	5.00 ± 1.04	5.12 ± 2.64	0.358

VAS: visual analog scale, SG: perceptive rehabilitation group, TG: rehabilitation group, and CG: control group.

**Table 2:** Mean ± standard deviation of clinical scores for the three groups for the three assessment times. *p* values in the last column for each scale refer to within-group Friedman analysis, whereas the *p* values in the lower rows refer to group comparisons based on Kruskal-Wallis analysis, with upper and lower bounds of the 95% confidence interval (95% CI). *p* values are reported in bold if statistically significant. Effect size is reported in the last rows for the comparisons between groups.

Scale	FIQ			*p* value (FR)	FAS			*p* value (FR)	HAQ			*p* value (FR)
Time of assessment	T0	T1	T2		T0	T1	T2		T0	T1	T2	
PS-group	68.0 ± 13.0	56.0 ± 13.0	55.0 ± 14.0	**<0.001**	7.1 ± 1.2	5.8 ± 1.3	5.5 ± 1.6	**<0.001**	1.1 ± 0.5	1.0 ± 0.6	0.9 ± 0.6	0.846
PE-group	66.0 ± 13.0	54.0 ± 10.0	54.0 ± 11.0	**0.003**	7.2 ± 1.3	6.0 ± 0.8	5.8 ± 0.8	**0.003**	1.3 ± 0.6	1.0 ± 0.6	0.9 ± 0.7	**<0.001**
CG	64.0 ± 9.0	66.0 ± 10.0	66.0 ± 10.0	**0.002**	7.4 ± 1.2	7.0 ± 1.3	7.0 ± 1.4	0.128	1.3 ± 0.4	1.5 ± 0.4	1.6 ± 0.4	**0.015**

*p* values (KW)	0.329	**0.013**	**0.007**		0.865	**0.004**	**0.004**		0.282	**0.009**	**0.001**	

95% CI												
UB	0.320	**0.010**	**0.004**		0.847	**<0.001**	**<0.001**		0.235	**0.001**	**<0.001**	
LB	0.380	**0.026**	**0.016**		0.889	**0.004**	**0.005**		0.289	**0.011**	**0.005**	

ES												
PS-group and CG	0.409	−0.839	−0.970		−0.251	−0.929	−1.007		−0.599	−1.077	−1.263	
PE-group and CG	0.176	−1.173	−1.185		−0.157	−0.938	−1.013		0.003	−0.992	−1.191	
PS-group and PE-group	−0.200	0.084	0.523		0.084	0.191	0.253		0.523	0.084	−0.022	

FIQ: Fibromyalgia Impact Questionnaire, FAS: Fibromyalgia Assessment Status, HAQ: Health Assessment Questionnaire, PS-group: perceptive surfaces group, PE: physical exercises group, CG: control group, T0: baseline, T1: end of the treatment, T2: follow-up, FR: Friedman analysis, KW: Kruskal-Wallis analysis, UB and LB: upper and lower bounds ES, and Cohen's *d*: effect size.

## Abbreviations

ACR: American College of Rheumatology
CAM: Complementary and alternative medicine
CG: Control group
CNS: Central nervous system
FAS: Fibromyalgia Assessment Status
FIQ: Fibromyalgia Impact Questionnaire
FM: Fibromyalgia
HAQ: Health Assessment Questionnaire
HR: Heart rate
PE: Physical exercises
PS: Perceptual surfaces
SAPS: Self-Assessment Pain Scale
PS-group: Group treated with perceptual surfaces
PE-group: Group treated with physical exercises
TPs: Tender points.
